# A high-performance voltammetric sensor based on Co/Ni-metal-organic framework modified electrode for the determination of dopamine in the presence of tyrosine

**DOI:** 10.5599/admet.3246

**Published:** 2026-05-07

**Authors:** Dhurgham Hani Kadhim Alalwan, Saja Haider Fadhil, Muntaha Mahmood Abed, Noor Kareem Aead, Hussein Ali Qabel

**Affiliations:** 1 Department of Pharmaceutical Chemistry, College of Pharmacy, University of Kerbala, Karbala, Iraq; 2 Department of Clinical Laboratory Sciences, College of Pharmacy, University of Kerbala, Karbala, Iraq

**Keywords:** Disposable sensor, differential pulse voltammetry, screen printed electrode, chronoamperometry, cyclic voltammetry, real sample analysis

## Abstract

**Background and purpose:**

The central nervous, renal, hormonal, and cardiovascular systems depend on dopamine. Therefore, a straightforward, sensitive, and selective method for detecting DA is needed to track DA levels in the human body.

**Experimental approach:**

Co/Ni-metal-organic framework was developed via a one-pot hydrothermal synthesis. The electrochemical sensor developed for dopamine measurement used Co/Ni-metal-organic framework materials on screen-printed carbon electrodes. The Co/Ni-metal-organic framework on a screen-printed carbon electrode exhibits excellent electrocatalytic activity for dopamine oxidation, owing to its high electron-transfer rate.

**Key results:**

The electrocatalytic activity of Co/Ni-metal-organic framework on a screen-printed carbon electrode significantly improves dopamine oxidation, yielding higher peak currents and lower oxidation potentials than the bare screen-printed carbon electrode. The sensor detected dopamine with a linear response, ranging from 0.01 to 660.0 μmol L^-1^, with a limit of detection of 0.007 μmol L^-1^. To measure tyrosine and dopamine simultaneously, differential pulse voltammetry was used. The separation between tyrosine and dopamine reached 150 mV.

**Conclusion:**

Successful target-analyte identification using the proposed voltammetric sensor was achieved, detecting both dopamine and tyrosine in actual sample tests.

## Introduction

The central nervous, renal, hormonal, and cardiovascular systems depend on dopamine (4-(2-aminoethyl)benzene-1,2-diol, DA), one of the most important neurotransmitters [1,2]. It also has a significant role in regulating cognitive functions, including behaviour, stress, and focus. DA is present as an organic cation in body fluids and brain tissues. Low dopamine levels are common in patients with Parkinson's disease due to the death of dopamine-containing neurons in the midbrain. Abnormal DA concentrations are also associated with schizophrenia, Huntington's disease, attention deficit hyperactivity disorder, and restless legs syndrome [3-5]. Therefore, a straightforward, sensitive, and selective method for detecting DA is needed to track DA levels in the human body.

Tyrosine, an essential amino acid involved in many bodily functions, is sometimes referred to as 4-hydroxyphenylalanine (Tyr). It is crucial for regulating protein synthesis. Tyr helps maintain the body's nitrogen balance. Tyr is present in foods, drugs, and dietary supplements [6,7]. Since phenylalanine cannot produce tyrosine without phenylalaninase, the production of phenylpyruvic acid, the secondary metabolic product, is greatly increased. Because of its effects on neurotransmitters, Tyr is used to treat conditions such as growth hormone stimulation, appetite suppression, and mood enhancement. Furthermore, studies show that abnormal tyrosine concentrations are directly associated with a number of human illnesses. For example, high Tyr levels may cause Parkinson's disease and enhanced sister chromatid exchange. The development of inborn disorders, including hawkininuria, tyrosinemia I, II, and III, and alkaptonuria, also depends on Tyr, a dopamine precursor. Furthermore, the development of type-2 diabetes, liver cancer, and obesity is significantly influenced by the concentration level of Tyr [8-10].

In the pharmaceutical industry, enzymatic processes can convert Tyr into DA [11] and it is important that Tyr and DA are measured at the same time in biological fluids. The methods reported for determining DA and Tyr include chemiluminescence [12,13], spectrophotometry [14,15], gas chromatography [16,17], and high-performance liquid chromatography [18,19]. These methods sometimes require multi-step sample preparation, despite their reputation for excellent accuracy. They should be handled appropriately because they also have certain disadvantages, including higher cost and greater time commitment. When it comes to DA and Tyr estimations, electrochemical methods were superior despite the wide range of other analytical techniques available [20,21].

Electrochemical methods have attracted significant interest for their inherent advantages, including rapid response times, high detection capabilities, user-friendly operation, non-toxicity, and the potential to create smaller devices. The techniques reduce analytical time and costs by handling samples without complex preparatory work and enabling analysis without it [22-25]. The adsorption of oxidized compounds leads to bare electrode fouling, reducing electrode performance and stability across multiple testing cycles. The direct electrochemical oxidation of DA and Tyr at bare electrodes requires substantial overpotential. The detection limit increases due to this factor, while the background current rises. The research results demonstrate that electroanalytical methods achieve improved performance with chemically modified electrode systems, which researchers use to create electrochemical sensors [26-30].

Electrochemical sensor development enables researchers to create devices that enable fast electron transfer while maintaining high selectivity through advances in nanomaterials. The scientific community has renewed its interest in nanomaterials due to their numerous applications in nanosensor development and other fields. Their special characteristics include a combination of large surface area and catalytic, mechanical and electrical properties [31-34].

All equipment needed for electrochemical analysis is portable, enabling screen-printed electrode (SPE)-based electrochemical sensors to function as in situ screening devices. The special features of screen-printed electrodes, which include compact size, low detection limit, fast reaction time and high repeatability, have made the technology popular for analytical use [35-38].

Metal-organic frameworks (MOFs) are extremely porous materials composed of metal cations linked by organic linkers. The extensive porosity and large internal surface area of MOFs, together with their tuneable pore structure and distinct active sites, enable their application in gas storage operations and separation processes, catalytic functions and the new field of electrochemical sensor detection [39-41]. The low electrical conductivity of the material restricts its development and application in electrochemical sensing technologies. The use of MOFs as direct electrode materials remains challenging.

Researchers have developed multiple techniques to achieve outstanding electrical conductivity in MOFs. The formation of bimetallic MOFs by partially replacing the primary metal with a secondary metal is an effective and straightforward method to enhance MOF conductivity. The electrical conductivity and charge-conduction properties of bimetallic MOFs can be controlled by adjusting the metal ratio in the material [42-45].

The researchers developed Co/Ni-MOF electrode materials through their basic method. The researchers used field-emission scanning electron microscopy (FE-SEM) to examine the material's surface structure. The researchers created a Co/Ni-MOF-modified electrode (Co/Ni-MOF/SPCE) by drop-casting a Co/Ni-MOF suspension onto the SPCE surface. The researchers used various electrochemical methods to evaluate the sensor's performance and optimized the detection conditions for DA. The produced electrode demonstrated excellent linearity over the range of 0.01 to 660.0 μmol L^-1^. The Co/Ni-MOF/SPCE sensor determined DA levels in the presence of Tyr, yielding two distinct voltammetric peaks for DA and Tyr.

The Co/Ni-MOF/SPCE sensor demonstrates effective performance for detecting both DA and Tyr in authentic sample analysis.

## Experimental

### Apparatus and chemicals

The general-purpose electrochemical system software operated the Autolab potentiostat/galvanostat, which conducted electrochemical experiments through its PGSTAT 302N instrument. The screen-printed electrode (DropSens, DRP-110, Spain) consists of an unmodified carbon working electrode, a silver pseudo-reference electrode and a carbon counter electrode. The Metrohm 710 pH meter was used to measure the pH.

All reagents and solvents met analytical quality standards. Tyrosine and dopamine were supplied by Merck (Darmstadt, Germany) without alteration. Phosphate buffer solutions (PBS) were prepared using orthophosphoric acid over the pH range 2.0 to 9.0.

### Synthesis of Co/Ni-MOF

**Figure 1.**
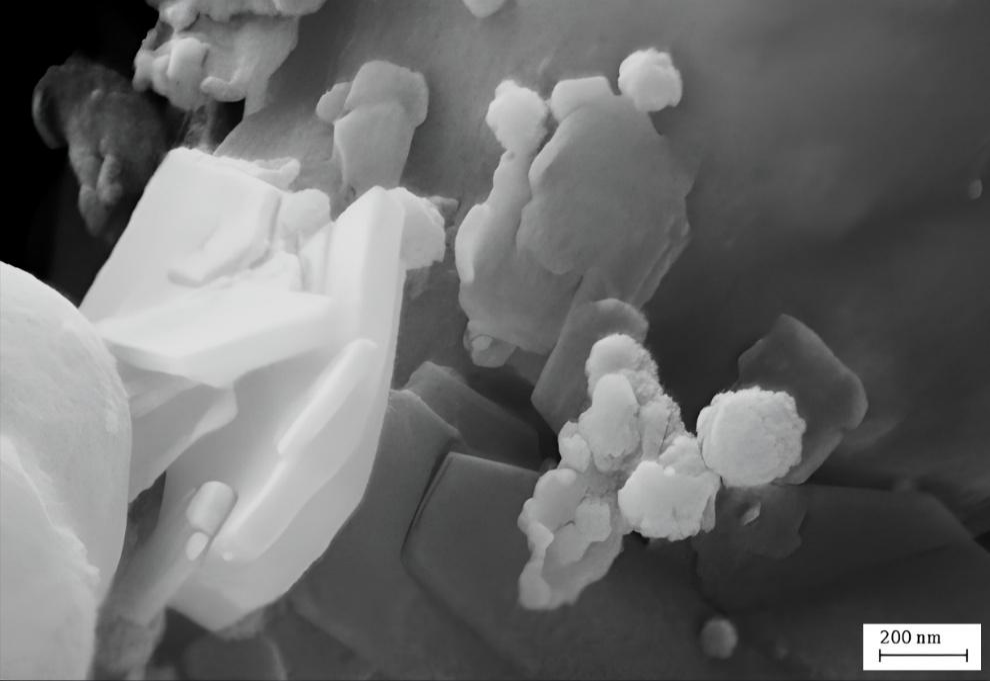
FE-SEM image of Co/Ni-MOF

Co(NO_3_)_2_·6H_2_O (1.2 mmol), NiCl_2_·6H2O (4 mmol), and terephthalic acid (1 mmol) were dissolved in dimethyl formamide (DMF) during a 10-minute period at room temperature while magnetic stirring. The autoclave contained the mixture during its operation. The system was heated to 120 °C for 16 hours, then allowed to cool naturally to ambient temperature. The centrifuge operation separated the products after five minutes at 9000 rpm and the materials were cleaned through multiple ethanol and DMF washings. The samples were dried under vacuum for 12 hours at 80 °C. Figure 1 displays the Co/Ni-MOF FE-SEM image.

### Co/Ni-MOF modified-SPCE

Co/Ni-MOF was applied to the modification of the SPCE in the following manner: a 4 μL aliquot of the Co/Ni-MOF/H_2_O suspension was cast onto the carbon working electrodes and allowed to evaporate at room temperature after a stock solution of Co/Ni-MOF in 1 mL of aqueous solution was prepared by dispersing 1 mg of Co/Ni-MOF with ultrasonication for 45 minutes. Ultimately, a modified electrode, Co/Ni-MOF/SPCE, was created. The surface area of the modified electrode was calculated to be 0.091 cm^2^ compared to 0.0318 cm^2^ for bare SPE, using the Randles-Ševčík equation.

## Results and discussion

### Electrochemical behaviour of dopamine at the Co/Ni-MOF/SPCE

One of the primary experimental factors influencing the voltammetric response of DA at Co/Ni-MOF/SPCE is the pH of the supporting electrolyte. Therefore, to enhance the strength of redox peak currents, an optimal pH is required. Therefore, using the differential pulse voltammetry (DPV) technique, the voltammetric responses of DA in 0.1 mol L^-1^ PBS were examined over the pH range from 2.0 to 9.0. The results show that the current signal of DA at Co/Ni-MOF/SPCE increases with increasing pH from 2.0 to 7.0, then decreases as the pH rises further. As a result, pH 7.0 was determined to be the ideal pH for further research.

**Figure 2.**
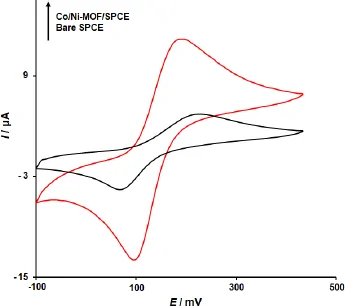
CV curves of 200.0 μmol L^-1^ DA in 0.1 mol L^-1^ PBS (pH 7.0) by bare SPCE and Co/Ni-MOF/SPCE at scan rates of 50 mV s^-1^

Cyclic voltammetry (CV) was used to study the electrochemical behaviour of DA on both the bare SPCE and a Co/Ni-MOF/SPCE in a 0.1 mol L^-1^, pH 7.0 PBS solution at a scan rate of 50 mV s^-1^ (Figure 2). The bare SPCE displays reversible behaviour for DA, as evidenced by small redox current peaks at *E*_pa_ (anodic peak potential) = 210 mV and *E*_pc_ (cathodic peak potential) = 75 mV. The Co/Ni-MOF/SPCE shows two distinct redox waves for DA, with *E*_pa_ at 180 mV and *E*_pc_ at 102 mV, as shown in Figure 2. The over-potential of DA decreases to less than that of the bare SPCE through an additional potential shift of 30 mV.

Also, the redox peak currents at Co/Ni-MOF/SPCE are higher than those at the bare SPCE. These results indicate that the modification of the SPCEs with Co/Ni-MOF greatly enhanced the electrochemical behaviour of the electrode for the analysis of DA.

### Influence of scan rate

**Figure 3.**
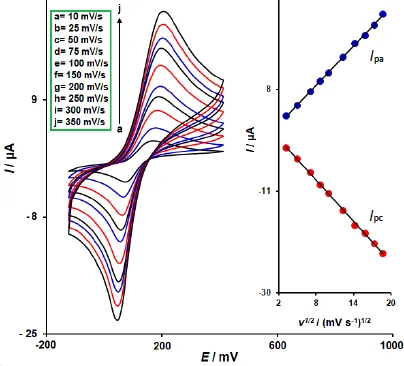
CV curves of 100.0 μmol L^-1^ DA in 0.1 mol L^-1^ PBS (pH 7.0) by Co/Ni-MOF/SPCE at various scan rates from 10 to 350 mV s^-1^. Insets: (A) linear relationship between *I*_pa_ and *v*^-1/2^; (B) linear relationship between *I*_pc_ and *v^-^*^1/2^

The study used CV to analyse how different scan rates (*v*) affected the oxidative and reductive peak currents of DA (100.0 μmol L^-1^) at Co/Ni-MOF/SPCE. The anodic and cathodic peak signals (*I*_pa_ and *I*_pc_), which were directly proportional to the scan rate over the range of 10 to 350 mV s^-1^, increased with the scan rate, as shown in Figure 3. The cathodic peak potentials shift in a negative direction, whereas the anodic peak potentials progressively move in a positive direction. The linear regression equation for the peak current and the square root of the scan rate (*v*^1/2^) is as follows: *I*_pa_ = 1.2215 *v* ^1/2^- 1.0459 (*R*^2^ = 0.9993) and *I*_pc_ = 1.2887*v* ^1/2^ + 1.3098 (*R*^2^ = 0.999). The results indicated that the electrochemical redox reaction of DA at the Co/Ni-MOF/SPCE was diffusion-controlled.

### Chronoamperometric studies

**Figure 4.**
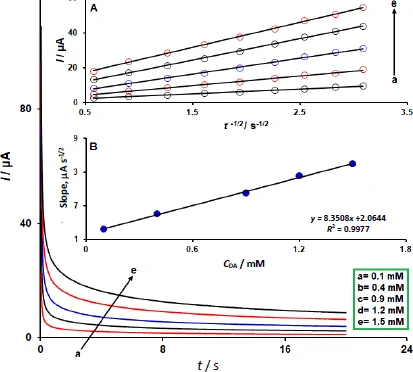
Chronoamperograms obtained at Co/Ni-MOF/SPCE in 0.1 mol L^-1^ PBS (pH 7.0) for different concentrations of DA from 0.1 to 1.5 mmol L^-1^. Insets: (A) plots of *I vs. t*^−1/2^ obtained from chronoamperograms; (B) plot of the slope of the straight lines against DA concentration

The working electrode potential was adjusted to 0.4 V for the different concentrations of DA (0.1 to 1.5 mmol L^-1^) in 0.1 mol L^-1^ PBS (pH 7.0) to perform chronoamperometric measurements of dopamine at Co/Ni-MOF/SPCE (Figure 4).

The Cottrell equation (*I = nFAC*_b_*D^1/2^π^-^*^1/2^*t^-^*^1/2^*)* describes the current measured for the electrochemical reaction at the mass transport-limited condition for an electroactive material (DA) with a diffusion coefficient of *D*. *I*_pa_ *vs. t*^−1/2^ experimental plots were used, and Figure 4A shows the best fits for various DA doses. Next, the slopes of the generated straight lines were plotted against DA concentration (Figure 4B). The mean value of the *D* was determined to be 5.95×10^-6^ cm^2^ s^-1^ based on the Cottrell equation and its associated slope.

### Quantitative measurements of dopamine at Co/Ni-MOF/SPCE sensor using DPV method

**Figure 5.**
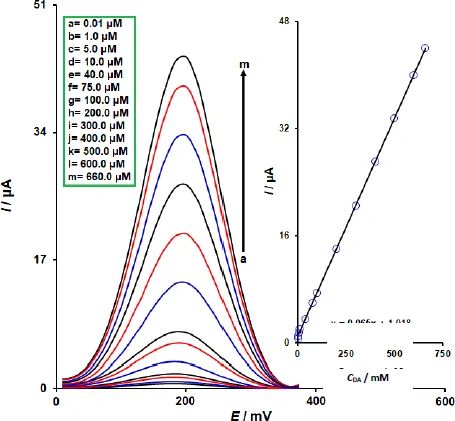
DPVs accepted *Co/Ni-MOF/SPCE* in PBS (0.1 mol L^-1^; pH 7.0) containing diverse concentrations of DA (from 0.01 to 660.0 μmol L^-1^). Inset: plot of the *I*_pa_ *vs.* DA concentrations

DA was oxidized at a Co/Ni-MOF/SPCE electrode in 0.1 mol L^-1^ PBS (pH 7.0) by adjusting its concentration between 0.01 and 660.0 μmol L^-1^. The plot of *I*_pa_ *vs.* DA concentration is shown in Figure 5 and indicates a linear relationship. It also indicates that peak current increases as DA concentration rises. *I*_pa_ = 0.065*C*_DA_ + 1.018 (*R* = 0.9999) is the linear fitted equation, and the linearity is found in the range of 0.01 to 660.0 μmol L^-1^. The LOD was computed using the formula: LOD = 3*S*_b_ *m*^-1^, where *m* is the calibration curve's slope and *S*_b_ is the blank's standard deviation. The LOD for the Co/Ni-MOF/SPCE sensor was 0.007 μmol L^-1^ based on the standard deviation of ten repeat readings (*n* = 10).

### The simultaneous determination of dopamine and tyrosine at Co/Ni-MOF/SPCE sensor

**Figure 6.**
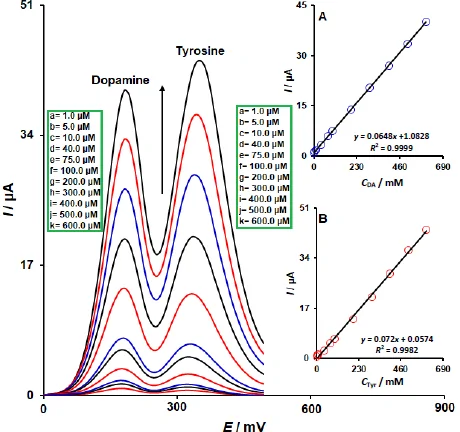
DPVs accepted Co/Ni-MOF/SPCE in PBS (0.1 mol L^-1^; pH=7.0) containing diverse concentrations of DA (from 1.0 μmol L^-1^ to 600.0 μmol L^-1^) and Tyr (from 1.0 μmol L^-1^ to 600.0 μmol L^-1^). Insets: A) plot of the I_pa_ versus. DA concentrations. B) Plot of the I_pa_ versus. Tyr concentrations

The simultaneous determination of DA and Tyr was the primary focus of this investigation. The DPVs for the DA and Tyr combination on Co/Ni-MOF/SPCE in 0.1 mol L^-1^ PBS (pH 7.0) are shown in Figure 6 after the concentrations of DA and Tyr were changed synchronously. With a peak difference of 150 mV, the current responses to the oxidation of Tyr (at 330 mV) and DA (at 180 mV) were shown to grow linearly, with *R*^2^ = 0.9982 and 0.9999, respectively. The Co/Ni-MOF/SPCE's sensitivity to the oxidation of DA in the presence of Tyr was found to be approximately 0.0648 μA L μmol^-1^, which was very close to the obtained value (0.065 μA L μmol^-1^) in the absence of Tyr. This suggests that the oxidation of these compounds on the Co/Ni-MOF/SPCE is independent, enabling simultaneous determination of their mixtures without significant interference.

### Application of the Co/Ni-MOF/SPCE platform for dopamine and tyrosine analysis in real sample

To assess the use of the Co/Ni-MOF/SPCE sensor for determining DA and Tyr in real samples (DA injection and urine), analytical experiments were conducted. Table 1 displayed the findings. Relative standard deviations (RSDs) ranged from 1.9 to 3.5 %, while recoveries ranged from 97.1 to 102.5 %. These findings show that the sensor developed in this work can detect DA and Tyr in real samples with high sensitivity and selectivity.

**Table 1.** Voltammetric determination of DA and Tyr in real specimens at Co/Ni-MOF/SPCE (*n*=5)

Sample	Added concentration, μmol L^-1^		Found concentration, μmol L^-1^		Recovery, %		RSD, %	
DA injection	DA	Tyr	DA	Tyr	DA	Tyr	DA	Tyr
	0	0	2.9	-	-	-	3.3	-
	2.0	5.0	4.8	5.1	98.0	102.0	2.7	2.6
	4.0	7.0	7.0	6.8	101.4	97.1	1.9	3.5
Urine	0	0	-	-	-	-	-	-
	5.5	6.0	5.6	5.9	101.8	98.3	3.4	2.1
	7.5	8.0	7.3	8.2	97.3	102.5	2.2	3.0

## Conclusions

An electrochemical sensing platform using Co/Ni-MOF materials was developed to measure dopamine after modification of SPCE. The Co/Ni-MOF materials demonstrate superior performance when combined with SPCE, yielding Co/Ni-MOF/SPCE with high electrocatalytic performance that enhances dopamine oxidation by reducing oxidation overpotentials and increasing peak current. The Co/Ni-MOF/SPCE sensor demonstrated a very low detection limit of 0.007 μmol L^-1^ and it could measure dopamine concentrations between 0.01 and 660.0 μmol L^-1^ through its linear detection capabilities. The Co/Ni-MOF/SPCE enabled researchers to identify DA and Tyr via DPV analysis because the method produced clear peak separation between the two compounds. The suggested approach has been successfully applied to measure DA and Tyr in real samples, which produced successful results.
